# Supplementary material for: Epidural Injection Method for Long-Term Pain Management in Rats with Spinal Stenosis
Source: Biomedicines. 2023 May 8;11(5):1390. doi: 10.3390/biomedicines11051390 (PMC10216675; doi:10.3390/biomedicines11051390)
Supplement: Supplementary file 1 [file biomedicines-11-01390-s001.zip › biomedicines-2359383-supplementary Figure S1.pdf]

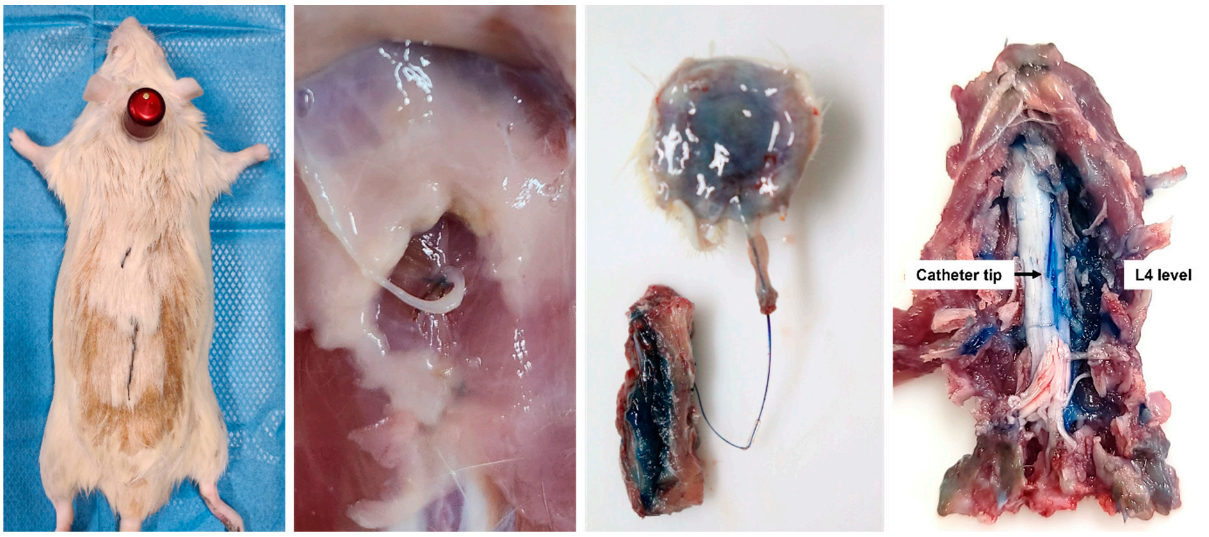

Figure S1. Confirmation of drug delivery device and catheter connection and positioning at the L4 level in a rat with lumbar spinal canal stenosis sacrificed at 3 weeks.
